# Editorial: Artificial intelligence for neuroimaging in the clinic - how compelling is the evidence?

**DOI:** 10.3389/fneur.2025.1576364

**Published:** 2025-03-04

**Authors:** Jennifer E. Soun, Brent D. Weinberg

**Affiliations:** ^1^Department of Radiological Sciences, University of California, Irvine, Irvine, CA, United States; ^2^Department of Radiology and Imaging Sciences, Emory University, Atlanta, GA, United States

**Keywords:** neuroimaging, artificial intelligence, radiomics, stroke, epilepsy

Artificial intelligence (AI) promises to play a major role in advancing neuroimaging techniques, but despite considerable interest and numerous publications on the topic, additional studies are needed before it becomes clinically impactful. This Research Topic explores a wide range of clinical applications of neuroimaging AI techniques, including temporal lobe epilepsy and vascular pathologies such as intracranial aneurysm, vertebrobasilar artery plaque, and vessel occlusion.

Epilepsy can be a challenging clinical diagnosis on imaging, as many patients can have normal imaging, and even when present, imaging abnormalities can be difficult to identify. Liao et al. applied a combination of radiomics and traditional machine learning (ML) techniques to distinguish patients with temporal lobe epilepsy (TLE) from healthy controls using 18F-FDG PET. The authors extracted radiomics features in the temporal regions most significantly associated with TLE and subsequently evaluated 11 ML algorithms to determine the best performing model in the training set. Logistic regression outperformed other ML algorithms, and the tuned logistic regression model demonstrated an AUC of 0.981 and 0.957 in training and test sets, respectively. Given this robust performance, this combined radiomics and ML approach may help the clinician in diagnosing TLE. A unique feature of this paper is its comparison of 11 different machine learning algorithms, as many papers in the field present a single approach only. This can be helpful to researchers who may have to select algorithms for future efforts.

The comprehensive mini-review by Wen et al. summarized the current landscape of AI and radiomics to study intracranial aneurysms. Wen et al. examined AI, radiomic, and combined AI-radiomic models for aneurysm detection, stability assessment, and outcome prediction, highlighting the performance and algorithms of these models. Challenges and limitations of these models, such as explainability, small aneurysm (<3 mm) detection, availability of high quality datasets, and generalizability in the clinical workspace, were acknowledged. This paper provides a high quality summary of the existing literature on this topic, providing a framework for future investigation in intracranial aneurysm evaluation.

Stroke is a common neuroradiology diagnosis, and advanced imaging techniques to identify patients at risk of future disease is a clear topic of interest. Liu et al. investigated a radiomics model to identify CT features of vertebrobasilary artery plaques which may contribute to posterior circulation strokes. The authors found that a radiomics model incorporating five selected features outperformed visual assessment of multiple calcifications, spotty calcification, and intimal predominant calcification to identify culprit plaques (AUC 0.81 for radiomics model vs. AUC 0.61–0.67 for visual assessment models). This manuscript highlights the utility of CT texture analysis in identifying imaging markers of plaque instability which may aid in risk stratification and management of patients with these lesions.

Han et al. also applied machine learning tools to help determine which stroke patients may benefit from intervention. These authors developed a deep learning algorithm to detect anterior circulation thrombectomy amenable vessel occlusions (TAVO). Deploying U-Net for vessel segmentation on maximum intensity projection CT angiography (CTA) images and EfficientNetV2 for TAVO prediction, the algorithm was able to detect TAVO with robust performance, demonstrating AUCs of 0.970 and 0.971 on two external datasets. Interestingly, the algorithm was able to detect isolated middle cerebral artery M2 occlusions with AUC 0.916 on combined external datasets. This was a novel aspect of the algorithm with important implications since M2 occlusions are increasingly being treated by thrombectomy but can be challenging for radiologists to manually identify.

In conclusion, these studies show the clinical applicability of utilizing AI in neuroimaging to improve our diagnosis and management of various CNS pathologies. These studies highlight the fact that AI, particularly when combined with other techniques such as radiomics, can discern disease states with robust performance and accuracy. The evidence from these studies is compelling, demonstrating that AI can enhance diagnostic capabilities for the clinician, thereby aiding in optimizing treatment plans and improving patient outcomes. They also provide a guide for future study as we move into a new generation of AI assisted imaging.

